# Proteinuria as the cause of early dose modification in Japanese patients with metastatic colorectal cancer treated with fruquintinib: a multicenter real-world study

**DOI:** 10.1007/s10147-026-03181-1

**Published:** 2026-08-27

**Authors:** Shohei Udagawa, Chihiro Ishizuka, Hiroki Osumi, Toshiharu Hirose, Natsuko Okita, Hidekazu Hirano, Hirokazu Shoji, Ken Kato, Koichiro Yoshino, Keitaro Shimozaki, Shota Fukuoka, Takeru Wakatsuki, Mariko Ogura, Akira Ooki, Keisho Chin, Kensei Yamaguchi, Atsuo Takashima, Eiji Shinozaki

**Affiliations:** 1 Department of Gastroenterological Chemotherapy, Cancer Institute Hospital of the Japanese Foundation for Cancer Research, Ariake 3-8-3, Koto-ku, Tokyo, Japan; 2 Department of Gastrointestinal Medical Oncology, National Cancer Center Hospital, Tokyo, Japan; 3 Department of Gastroenterology, Kanagawa Cancer Center, Yokohama, Japan

**Keywords:** Fruquintinib, Metastatic colorectal cancer, Real-world study, Dose modification, Proteinuria

## Abstract

**Background:**

Fruquintinib has demonstrated survival benefits in patients with previously treated metastatic colorectal cancer (mCRC) in phase III trials. However, real-world data in Japanese patients remain limited. This study evaluated the effectiveness and safety of fruquintinib in routine clinical practice.

**Methods:**

We conducted a retrospective observational study at two cancer centers. Patients treated with fruquintinib for mCRC between November 2024 and December 2025 were included. Clinical outcomes and safety were evaluated, and exploratory analyses were performed according to starting dose, relative dose intensity (RDI), and selected adverse events (AEs).

**Results:**

A total of 92 patients were included. Fruquintinib was initiated at 5 mg in 72 patients (78.3%) and at 4 mg in 19 patients (20.7%). The disease control rate was 37.4%, and no objective responses were observed. Median time to treatment failure was 2.57 months (95% confidence interval [CI], 2.23–3.33), and median overall survival was 8.83 months (95% CI, 7.46–not estimable). The median follow-up period was 5.48 months. Grade ≥ 3 AEs occurred in 39 patients (42.4%), and treatment discontinuation due to AEs occurred in 4 patients (4.3%). Proteinuria was the leading cause of early dose reduction, with 39.1% of dose modifications occurring within 28 days. In exploratory analyses, no clear differences in outcomes were observed according to starting dose, RDI, hypertension, or proteinuria.

**Conclusions:**

Fruquintinib demonstrated feasible effectiveness and manageable toxicity in Japanese patients with mCRC. Proteinuria was a major determinant of treatment feasibility and frequently led to early dose modification.

**Supplementary Information:**

The online version contains supplementary material available at https://doi.org/10.1007/s10147-026-03181-1.

## Introduction

Colorectal cancer (CRC) is a major global public health concern. It is the third most commonly diagnosed cancer worldwide, with approximately 1.9 million new cases (9.6% of all cancers), and the second leading cause of cancer-related death, accounting for approximately 900,000 deaths [1]. Approximately 15–25% of patients with CRC present with distant metastases at the time of diagnosis [2]. The prognosis of metastatic CRC (mCRC) remains poor, with a 5-year relative survival rate of approximately 14% worldwide [2]. According to the 2023 estimates in Japan, CRC is the second most common cancer, with approximately 159,000 new cases [3]. It is also a leading cause of cancer-related death in Japan, ranking second in men and first in women, with a total of approximately 54,000 deaths annually [3]. 

In recent years, particularly in the early-line setting, targeted therapies have been developed based on molecular characteristics and primary tumor location. In the later-line setting, several agents have shown survival benefits regardless of molecular subtype. Trifluridine/tipiracil, regorafenib, and fruquintinib have each shown significant improvements in survival compared with placebo or best supportive care in phase III trials and are now established treatment options [4–8]. In particular, the addition of bevacizumab to trifluridine/tipiracil has been shown to provide additional survival benefit, as demonstrated in the SUNLIGHT trial [9].

Among these agents, fruquintinib is the most recently introduced drug. Fruquintinib is a potent and highly selective oral small-molecule tyrosine kinase inhibitor targeting vascular endothelial growth factor receptors (VEGFR) 1, 2, and 3 [10]. Its efficacy has been demonstrated in phase III trials, including the FRESCO and FRESCO-2 studies, both of which showed significant improvements in overall survival (OS) and progression-free survival (PFS) compared with placebo in patients with previously treated mCRC [7, 8]. In the FRESCO-2 trial, a post hoc subgroup analysis in Japanese patients suggested similar efficacy compared with the overall population. However, a higher incidence of adverse events (AEs) was observed in Japanese patients [11, 12]. It should be noted that this analysis was based on a small sample size of only 40 patients treated with fruquintinib. In addition, clinical experience with fruquintinib remains limited, and real-world data, including those from Japanese patients, are still limited. Although several real-world studies have reported the overall effectiveness and safety of fruquintinib in China, clinically relevant questions regarding dose modification, dose intensity, and the practical management of AEs in routine practice remain insufficiently addressed [13–16]. In routine clinical practice, especially in the later-line setting, patients often have poorer general conditions than those enrolled in clinical trials. Therefore, this retrospective study aimed to evaluate the real-world effectiveness and safety of fruquintinib in patients with mCRC in Japan, with a particular focus on treatment-related AEs and dose modification in routine clinical practice.

## Methods

### Study design

We conducted a retrospective multicenter observational study at the Cancer Institute Hospital of the Japanese Foundation for Cancer Research and National Cancer Center hospital in Japan. The inclusion criteria were (1) diagnosis of mCRC, (2) histologically confirmed adenocarcinoma, and (3) treatment with fruquintinib between November 2024 and December 2025. The data cut-off date for follow-up was February 2026. Fruquintinib was administered according to the approved prescribing information and institutional guidelines. Dose modifications were performed at the discretion of the treating physicians based on patient condition and AEs. The study protocol (2024-GB-072) was approved by the Ethics Committee of Cancer Institute Hospital of the Japanese Foundation for Cancer Research procedures were conducted in accordance with the ethical standards of the Institutional and National Research Committee and the 1964 Declaration of Helsinki and its later amendments. The requirement for informed consent was waived due to the retrospective design of the study. Information regarding the study was disclosed to the public, and an opt-out approach was employed in accordance with the institutional guidelines.

## Data collection

All data were obtained through medical record review. Complete response (CR), partial response (PR), stable disease (SD), and progressive disease (PD) were defined according to Response Evaluation Criteria in Solid Tumors (RECIST) guidelines (v1.1). Time to treatment failure (TTF) was defined as the time from the initiation of fruquintinib to treatment discontinuation for any reason, including disease progression, AEs, or death from any cause. OS was defined as the time from fruquintinib initiation to death from any cause. Each AE was evaluated according to the Common Terminology Criteria for Adverse Events (ver. 5.0). Proteinuria was mainly assessed using urine dipstick testing. When proteinuria of ≥ 2 + was detected, the urine protein-to-creatinine ratio was additionally measured. During the first treatment cycle, patients were generally reassessed once at 1 or 2 weeks after the initiation of fruquintinib. Relative dose intensity (RDI) was evaluated in patients who received fruquintinib for at least 2 months to reduce potential bias from very short treatment duration. RDI was calculated as the ratio of the actual cumulative dose to the planned cumulative dose and expressed as a percentage. The planned cumulative dose was based on the standard regimen of fruquintinib 5 mg once daily for 3 weeks followed by 1 week off, which was defined as 100% dose intensity. Estimated glomerular filtration rate (eGFR) was calculated using the Japanese equation based on serum creatinine, age, and sex, and was expressed in mL/min/1.73 m^2^.

## Statistical analyses

Categorical variables were compared using Fisher’s exact test, and continuous variables were compared using Student’s t-test. TTF and OS were estimated using the Kaplan–Meier method, and the results were compared using the log-rank test. A Cox proportional hazards model was used to estimate hazard ratios (HRs) and 95% confidence intervals (CIs). Variables considered clinically relevant were selected a priori with consideration of the number of events and included in the multivariable Cox regression models regardless of their significance in the univariable analyses. Exploratory subgroup analyses for TTF and OS were performed according to the starting dose of fruquintinib, the worst grade of selected AEs, and RDI. For the RDI analysis, patients were categorized into low- and high-RDI groups according to the median RDI value. All *P*-values were obtained using two-tailed tests. Statistical significance was defined as *P* < 0.05. All statistical analyses were performed using EZR version 1.41 statistical software (Saitama Medical Center, Jichi Medical University, Saitama, Japan), which is a graphical user interface for R (The R Foundation for Statistical Computing, Vienna, Austria) [17].

## Results

### Patient characteristics

A total of 92 patients with mCRC who received fruquintinib were included in this study. Baseline patient characteristics are summarized in Table 1. The median age was 61.5 years (range, 29–87), and 49 patients (53.3%) were male. ECOG performance status was 0–1 in 88 patients (95.6%) and 2 in 4 patients (4.4%). The median number of previous treatment lines was 4, and all patients had previously received trifluridine–tipiracil. Thirty-two patients (34.8%) had also previously received regorafenib. The median duration of metastatic disease was 36.2 months (IQR, 22.8–59.6). The starting dose of fruquintinib was 5 mg in 72 patients (78.3%), 4 mg in 19 patients (20.6%), and 3 mg in 1 patient (1.1%). No patients who started at 4 mg underwent subsequent dose escalation to 5 mg. Among the 20 patients who started treatment at a reduced dose, the documented reasons were older age in 6 patients (30.0%), proteinuria in 4 patients (20.0%), ECOG performance status 2 in 3 patients (15.0%), poorly controlled hypertension in 2 patients (10.0%), and interstitial lung disease, patient preference, hemoptysis, history of radiotherapy, history of intracranial hemorrhage, and unknown reason in 1 patient each (5.0%). The median follow-up period was 5.48 months (IQR, 3.93–7.91), and the median RDI was 69.8% (IQR, 57.1–84.3). After discontinuation of fruquintinib, 28 patients (30.4%) received subsequent treatment. These treatments included regorafenib in 5 patients (5.4%), rechallenge with previously used agents in 15 patients (16.3%), and other treatments in 8 patients (8.7%).

**Table 1:** Patient characteristics

	Total *n* = 92 (%)
Age, years	
Median (range)	61.5 (29–87)
≥ 65 years	37 (40.2)
Sex	
Female	43 (46.7)
Male	49 (53.3)
Performance status	
0	37 (40.2)
1	51 (55.4)
2	4 (4.4)
Primary location	
Right colon	24 (26.1)
Left colon	36 (39.1)
Rectum	32 (34.8)
*RAS* / *BRAF* status	
*RAS* mutant	50 (54.3)
*BRAF* mutant	5 (5.4)
Wild type or unknown	37 (40.2)
MSI status	
MSS/pMMR	92 (100)
MSI-H/dMMR	0 (0)
Previous anti-EGFR monoclonal antibody therapy	
Yes	38 (41.3)
No	54 (58.7)
Number of previous treatment lines	
2	5 (5.4)
3	36 (39.1)
4	26 (28.3)
≥ 5	25 (27.2)
Duration of metastatic disease	
Median (IQR), month	36.2 (22.8–59.6)
Metastatic sites	
Liver	53 (57.6)
Lung	67 (72.8)
Previous anti-VEGF monoclonal antibody	86 (93.5)
Previous trifluridine–tipiracil or regorafenib	
Trifluridine–tipiracil	92 (100)
Concomitant bevacizumab	78 (84.8)
Regorafenib	32 (34.8)
Both	32 (34.8)
Starting dose of Fruquintinib	
5 mg	72 (78.3)
4 mg	19 (20.6)
3 mg	1 (1.1)
Subsequent treatment	
None	64 (69.6)
Regorafenib	5 (5.4)
Rechallenge with previously agents	15 (16.3)
Others treatment	8 (8.7)

BRAF, v-Raf murine sarcoma viral oncogene homolog B; dMMR, deficient mismatch repair; EGFR, epidermal growth factor receptor; IQR, interquartile range; MSI-H, microsatellite instability-high; MSS, microsatellite stable; pMMR, proficient mismatch repair; RAS, rat sarcoma viral oncogene homolog; VEGF, vascular endothelial growth factor

### Treatment efficacy

**Fig. 1:**
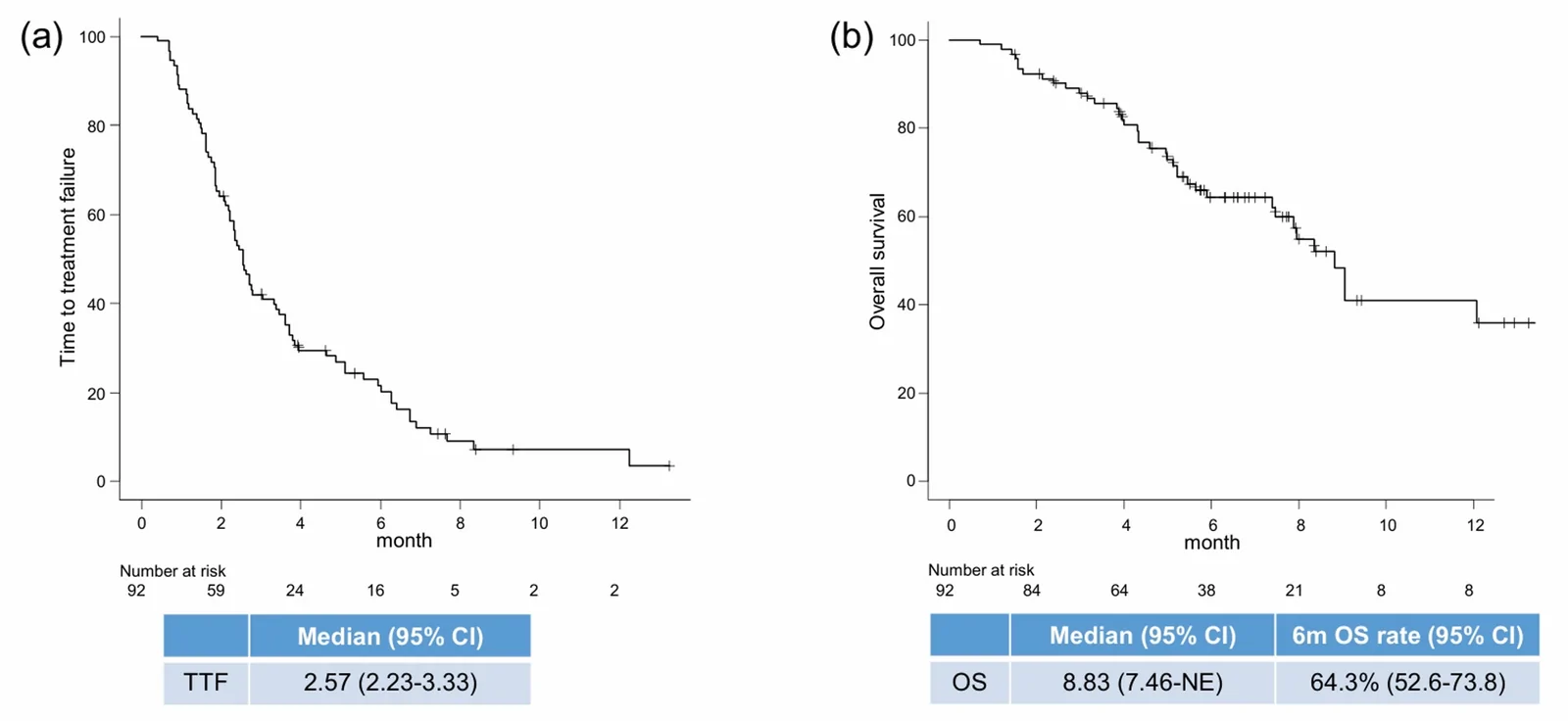
Kaplan–Meier curves for **a** time to treatment failure and **b** overall survival. CI confidence interval, OS overall survival, TTF time to treatment failure

The median TTF was 2.57 months (95% CI, 2.23–3.33), and the median OS was 8.83 months (95% CI, 7.46–NE). The 6-month OS rate was 64.3% (95% CI, 52.6–73.8) (Fig. 1). Tumor response was evaluable in 83 patients. At the data cutoff, 38 deaths were observed among the 92 patients included in the OS analysis. No CR or PR were observed. SD was observed in 31 patients (37.4%), and progressive disease in 45 patients (54.2%). Seven patients (8.4%) were not evaluable. The response rate was 0%, and the disease control rate was 37.4% (Table 2). In the univariate analysis for TTF, the HR for age > 65 years was 0.59 (95% CI, 0.37–0.93; *P* = 0.02), and the HR for liver metastases was 1.94 (95% CI, 1.19–3.15; *P* = 0.01). In the multivariable analysis for TTF, the HR for age > 65 years was 0.57 (95% CI, 0.34–0.96; *P* = 0.03), and the HR for liver metastases was 2.09 (95% CI, 1.29–3.38; *P* < 0.01). In the multivariate analysis for OS, none of the evaluated clinical factors were significantly associated with OS. The results of the univariate and multivariable Cox proportional hazards analyses for TTF and OS are summarized in Table 3.

**Table 2:** Tumor response in patients with target lesion

Tumor response	*n *= 83 (%)
Complete response	0
Partial response	0
Stable disease	31 (37.4)
Progressive disease	45 (54.2)
Not evaluable	7 (8.4)
Disease control rate	31 (37.4)
Response rate	0

**Table 3:** Cox proportional hazard analysis for factors associated with TTF and OS

TTF	Univariate analysis		Multivariable analysis	
	HR (95% CI)	*P* value	HR (95% CI)	*P* value
Sex (Male* or Female)	1.16 (0.75–1.79)	0.51	1.53 (0.95–2.45)	0.08
Age (≤ 65* or > 65 years)	0.59 (0.37–0.93)	0.02	0.57 (0.34–0.96)	0.03
Performance status (0* or 1, 2)	0.72 (0.45–1.13)	0.15	0.77 (0.47–1.25)	0.29
Primary location (Right* or Left)	0.91 (0.55–1.50)	0.71	0.97 (0.55–1.71)	0.92
*RAS/BRAF* mutant (No* or Yes)	1.08 (0.69–1.70)	0.74	1.21 (0.74–1.99)	0.44
Number of previous treatment lines (2,3* or ≥ 4)	0.84 (0.54–1.30)	0.42		
Liver metastases (No* or Yes)	1.94 (1.19–3.15)	0.01	2.09 (1.29–3.38)	< 0.01
Lung metastases (No* or Yes)	1.12 (0.66–1.89)	0.69		
Prior use of regorafenib (No* or Yes)	1.30 (0.80–2.12)	0.29	1.56 (0.92–2.63)	0.10
Starting dose of Fruquintinib (4, 3 mg* or 5 mg)	0.98 (0.56–1.71)	0.94	0.65 (0.36–1.18)	0.15

OS	Univariate analysis		Multivariable analysis	
	HR (95% CI)	*P* value	HR (95% CI)	*P* value
Sex (Male* or Female)	1.39 (0.73–2.63)	0.31	1.48 (0.74–2.93)	0.26
Age (≤ 65* or > 65 years)	0.87 (0.45–1.69)	0.68	0.75 (0.37–1.53)	0.43
Performance status (0* or 1, 2)	1.51 (0.77–2.97)	0.23	1.58 (0.77–3.23)	0.21
Primary location (Right* or Left)	1.03 (0.50–2.14)	0.94	0.95 (0.41–2.19)	0.91
*RAS/BRAF* mutant (No* or Yes)	1.12 (0.58–2.17)	0.74	1.29 (0.62–2.68)	0.49
Number of previous treatment lines (2,3* or ≥ 4)	0.95 (0.50–1.82)	0.88		
Liver metastases (No* or Yes)	1.59 (0.82–3.09)	0.17	1.76 (0.89–3.46)	0.10
Lung metastases (No* or Yes)	0.86 (0.42–1.73)	0.66		
Prior use of regorafenib (No* or Yes)	1.29 (0.67–2.47)	0.44	1.76 (0.83–3.75)	0.83
Starting dose of Fruquintinib (4, 3 mg* or 5 mg)	0.83 (0.38–1.83)	0.64	0.60 (0.26–1.41)	0.24

* Reference

BRAF, v-Raf murine sarcoma viral oncogene homolog B; CI, confidence interval; HR, hazard ratio; OS, overall survival; RAS, rat sarcoma viral oncogene homolog; TTF, time to treatment failure

## Safety

Treatment-related AEs are summarized in Table 4. The most frequent any-grade and Grade ≥ 3 AEs were hypertension (50.0% and 14.1%), proteinuria (44.6% and 22.8%), and hand-foot syndrome (HFS) (43.5% and 7.6%). Treatment discontinuation due to AEs occurred in 4 patients, and the reasons were anorexia and fatigue, proteinuria, HFS, and diarrhea. Dose reduction due to AEs occurred in 34 of 72 patients (47.2%) who started fruquintinib at 5 mg and in 10 of 19 patients (52.6%) who started at 4 mg. Among these patients, AEs requiring dose reduction or treatment interruption within 28 days were observed in 27 of 34 patients (79.4%) in the 5 mg group and 9 of 10 patients (90.0%) in the 4 mg group. The distribution of dose modifications from treatment initiation to Day 29 and to the last administered dose is illustrated in Online Resource 1. Proteinuria was the most frequent reason for dose reduction, followed by hypertension and HFS (Table 5). The incidences of proteinuria, hypertension, and HFS were also evaluated according to baseline proteinuria, maximum proteinuria during prior treatment, bevacizumab use within 1 month before fruquintinib initiation, use of antihypertensive medication at treatment initiation, and prior regorafenib treatment, respectively. No statistically significant differences were observed among these subgroups (Online Resource 2). Baseline factors associated with AEs leading to dose reduction are shown in Online Resource 3. Among patients who started at 5 mg, those who required dose reduction were older than those who did not (median age, 62.5 vs. 56.5 years; *P* = 0.01). Among patients who started at 4 mg, patients who required further dose reduction were older (76.5 vs. 62.0 years; *P* = 0.01), had lower body weight (50.5 vs. 67.0 kg; *P* = 0.02), and had lower eGFR (62.9 vs. 90.7 mL/min/1.73 m²; *P* = 0.03). Patients with Grade ≥ 3 proteinuria had a higher median age than those with Grade ≤ 2 proteinuria in both starting dose groups. Among patients who started at 5 mg, bevacizumab use within 1 month before fruquintinib initiation was more frequent in patients with Grade ≥ 3 proteinuria than in those with Grade ≤ 2 proteinuria (Online Resource 4). The overlap among proteinuria, hypertension, and HFS is shown in Online Resource 5.

**Table 4:** Adverse events

	*n* = 92(%)	
	Any grade	Grade ≥ 3
Hypertension	46 (50.0)	13 (14.1)
Proteinuria	41 (44.6)	21 (22.8)
Hand foot syndrome	40 (43.5)	7 (7.6)
Liver dysfunction	10 (10.9)	0
Platelet count decreased	6 (6.5)	4 (4.3)
Diarrhea	6 (6.5)	1 (1.1)
Skin rash	5 (5.4)	1 (1.1)
Hypothyroidism	4 (4.3)	0
Fatigue	3 (3.3)	0
Decreased appetite	2 (2.2)	0
Arthralgia	2 (2.2)	0

**Table 5:** Reason and Timing for AEs leading to dose reduction

	5 mg start *n* = 72		4 mg start *n* = 19
	4 mg *n* = 34	3 mg *n* = 5	3 mg *n* = 10
Within 28 days	27 (79.4)	–	9 (90.0)
Proteinuria	15 (44.1)	–	5 (50.0)
Hypertension	3 (8.8)	–	1 (10.0)
Hand foot syndrome	6 (17.6)	–	2 (20.0)
Platelet count decreased	4 (11.8)	–	0
Fatigue	2 (5.9)	–	0
Skin rash	1 (2.9)	–	1 (10.0)
Decreased appetite	1 (2.9)	–	0
Diarrhea	1 (2.9)	–	0
After 28 days	7 (20.4)	5 (100)	1 (10.0)
Proteinuria	4 (11.8)	2 (40.0)	0
Hypertension	1 (2.9)	2 (40.0)	0
Hand foot syndrome	1 (2.9)	0	0
CK elevated	1 (2.9)	0	0
Platelet count decreased	0	1 (20.0)	0
Skin rash	0	0	1 (10.0)

## Exploratory analyses of clinical factors associated with treatment outcomes

**Fig. 2:**
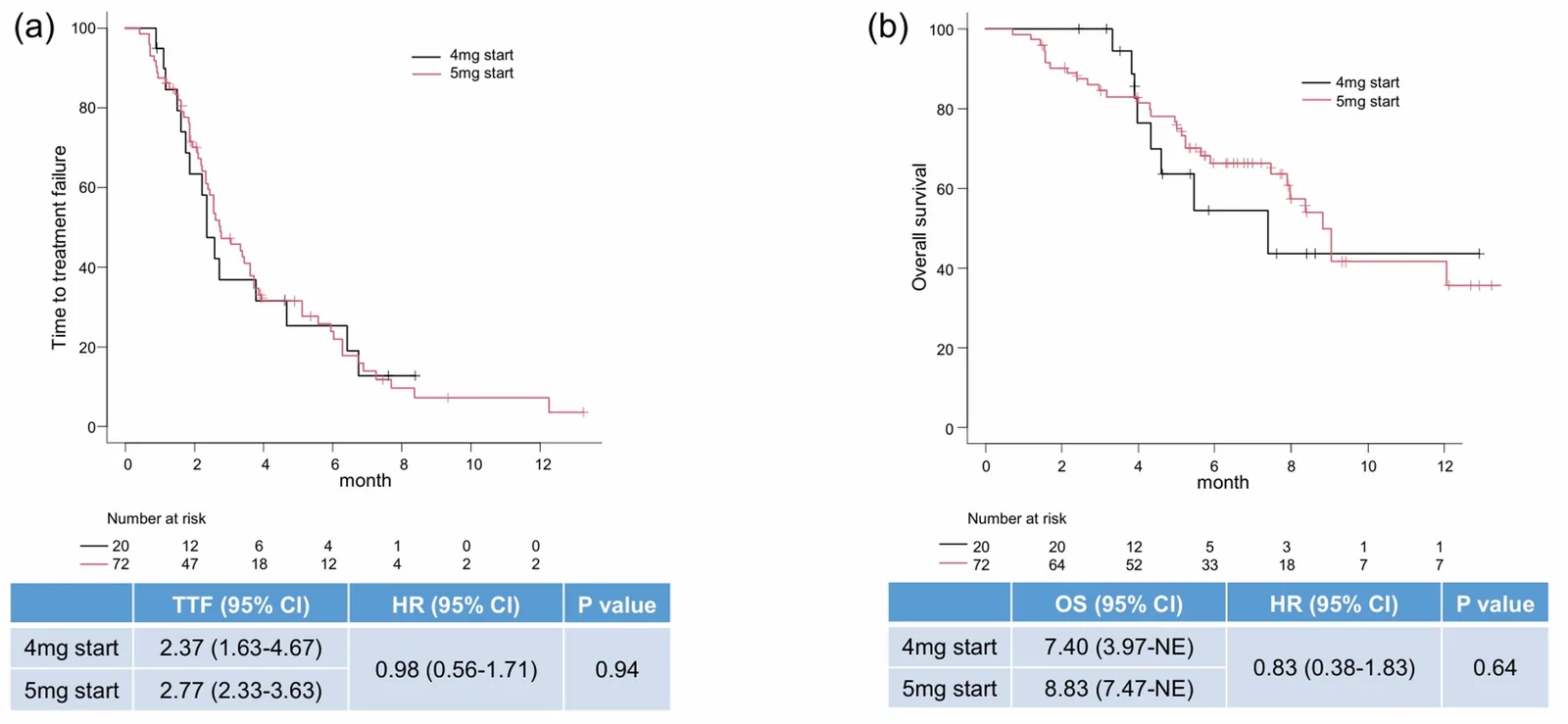
Kaplan–Meier curves for **a** time to treatment failure (TTF) and **b** overall survival (OS) stratified by the starting dose of fruquintinib (4 mg vs. 5 mg). CI, confidence interval; HR, hazard ratio; OS, overall survival; TTF, time to treatment failure

**Fig. 3:**
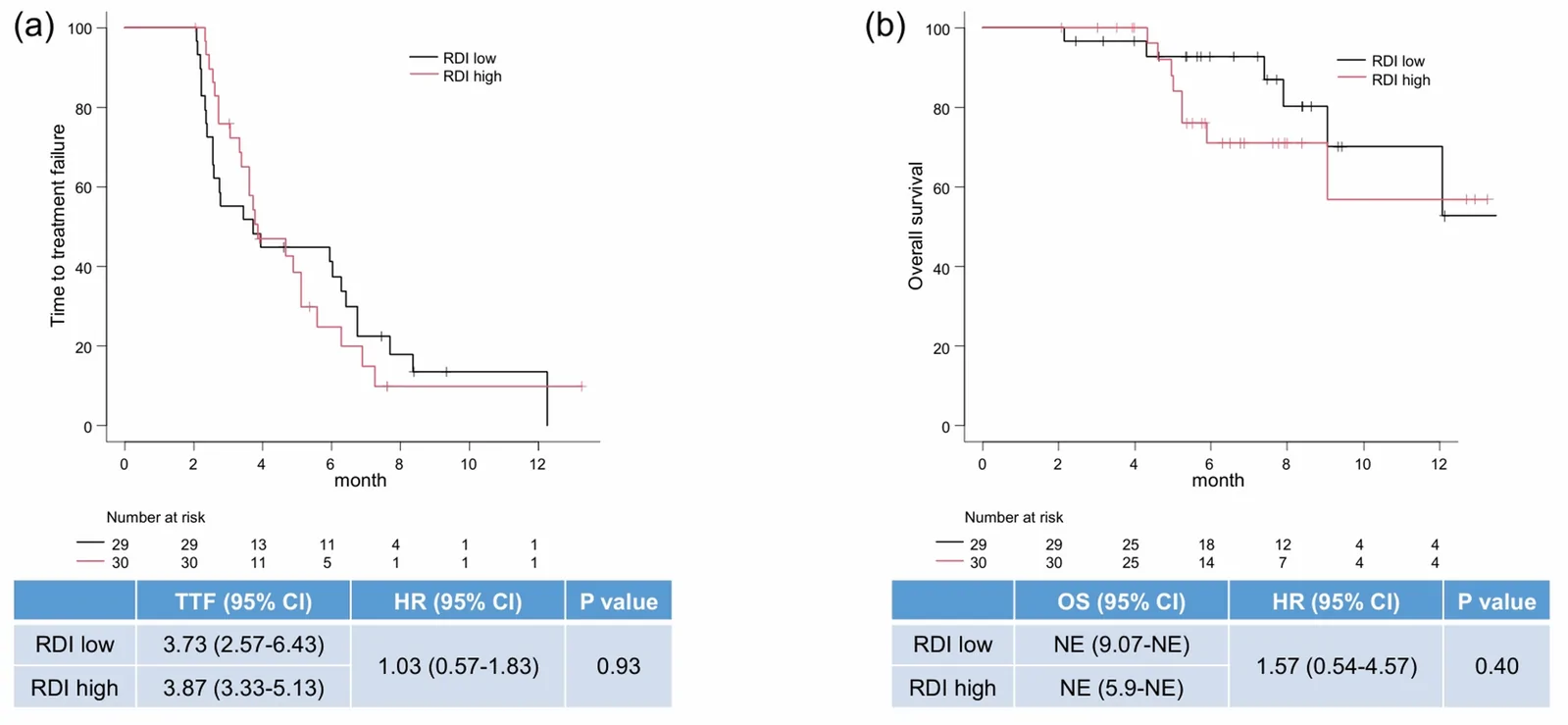
Kaplan–Meier curves for **a** time to treatment failure (TTF) and **b** overall survival (OS) stratified by relative dose intensity (RDI) (low vs. high), categorized according to the median RDI. CI, confidence interval; HR, hazard ratio; OS, overall survival; RDI, relative dose intensity; TTF, time to treatment failure

Exploratory analyses were performed to evaluate treatment outcomes according to starting dose, selected AEs, and RDI. For starting dose, the median TTF (2.77 vs. 2.37 months; HR 0.98, 95% CI 0.56–1.71; *P* = 0.94) and OS (8.83 vs. 7.40 months; HR 0.72, 95% CI 0.38–1.38; *P* = 0.33) were comparable between the starting dose of 5 mg and 4 mg groups (Fig. 2). Similarly, no significant differences in outcome were observed based on the severity of proteinuria(Grade ≤ 1 vs. ≥2) (TTF: 2.57 vs. 3.33 months, HR, 0.83; 95% CI 0.52–1.31; *P* = 0.41) (OS: 7.97 vs. 9.07 months, HR, 0.72; 95% CI 0.38–1.38; *P* = 0.33), hypertension (TTF: 2.73 vs. 2.60 months, HR, 0.93; 95% CI 0.59–1.47; *P* = 0.76) (OS: 8.37 vs. 8.83 months, HR, 1.00; 95% CI 0.52–1.88; *P* = 0.99) (Online Resources 6, 7). For HFS, the median TTF was 2.47 vs. 3.30 months (HR, 0.64; 95% CI, 0.38–1.08; *P* = 0.09) and the OS was 7.97 vs. NE months months (HR, 0.33; 95% CI, 0.12–0.94; *P* = 0.04) based on the severity of HFS (Grade ≤ 1 vs. ≥2). (Online Resource 8). Furthermore, RDI was not significantly associated with either TTF or OS. The median TTF was 3.87 months in the high-RDI group and 3.73 months in the low-RDI group (HR, 1.03; 95% CI, 0.57–1.83; *P* = 0.93; Fig. 3), while the median OS was not reached in either group (HR, 1.57; 95% CI, 0.54–4.57; *P* = 0.40) (Fig. 3).

## Discussion

In this study, fruquintinib demonstrated clinically meaningful activity and manageable toxicity in Japanese patients with mCRC in the later-line setting. The overall effectiveness observed in this real-world cohort was generally consistent with that reported in previous phase III trial [7, 8]. In addition, dose reduction during treatment was frequently required in routine clinical practice, with proteinuria emerging as the leading cause of early dose modification. Despite this, exploratory analyses showed no clear difference in treatment outcomes according to starting dose or RDI. These findings suggest that fruquintinib can be feasibly administered in routine clinical practice with appropriate dose adjustment.

Compared with the FRESCO and FRESCO-2 trials, the ORR and TTF in the present study were somewhat less favorable, whereas OS was generally comparable [7, 8]. This finding supports the clinical utility of fruquintinib in Japanese patients with mCRC in routine clinical practice. Several factors may explain the relatively lower response and shorter TTF observed in this cohort. First, patients treated in routine clinical practice are often more heterogeneous and may have poorer general conditions than those enrolled in prospective clinical trials. Second, in clinical practice, radiologic evaluation may be performed earlier than scheduled when clinical deterioration is suspected. These differences in patient selection and assessment timing may have influenced the ORR and TTF. In addition, because fruquintinib had only recently been approved in Japan just before the study period, some patients may have initiated treatment after waiting for its availability, potentially including those with more advanced disease or poorer general condition, which may have also contributed to the shorter TTF observed in this cohort. In the present study, TTF was used instead of PFS as a clinically relevant endpoint. In later-line treatment, it is often difficult to clearly distinguish whether worsening general condition is caused by disease progression or treatment-related toxicity. In addition, treatment discontinuation in routine clinical practice may be influenced by factors other than radiologic progression, including tolerability and patient preference. Therefore, TTF may represent a more practical endpoint than PFS for evaluating treatment continuation in real-world later-line settings.

In the present study, proteinuria emerged as a key determinant of treatment feasibility. Proteinuria, hypertension, and HFS were all frequently observed and contributed to dose reduction, with proteinuria being the most frequent cause of early dose reduction. This observation differs from the safety profile reported in the FRESCO-2 trial, in which proteinuria was recognized as a class-specific adverse event associated with VEGFR inhibition, with an incidence of 17.8% for any grade and 1.5% for Grade ≥ 3 events, and was generally considered manageable with standard monitoring and dose modification strategies [12]. However, its impact on treatment feasibility was not emphasized, and it was relatively infrequent as a cause of dose reduction [12]. In contrast, in our real-world cohort, proteinuria frequently required early dose modification, suggesting a greater impact on treatment continuation. Notably, a large proportion of AEs leading to dose reduction occurred within the first 28 days, which is consistent with pharmacovigilance data from the FDA Adverse Event Reporting System indicating that most fruquintinib-related adverse events occur within the first 30 days of treatment initiation [18]. These findings highlight the importance of early monitoring and management of toxicity in routine clinical practice. The discrepancy between our findings and those of the FRESCO-2 trial and real-world studies from China may reflect differences in patient populations and treatment exposure. The FRESCO-2 trial predominantly included Western patients, and ethnic differences in susceptibility to VEGF-related toxicities may partly contribute to the observed findings. In contrast, real-world studies from China have reported lower rates of prior VEGF inhibitor use (34.6%–64%), whereas 93.5% of patients in our cohort had received prior VEGF-targeted therapy [13, 16]. In the additional exploratory analysis, we evaluated proteinuria at treatment initiation and the maximum proteinuria during prior treatment, and anti-VEGF antibody use within 1 month before fruquintinib initiation. No clear associations were observed in the overall population. However, among patients who started fruquintinib at 5 mg, anti-VEGF antibody use within 1 month was more frequent in patients with Grade ≥ 3 proteinuria than in those with lower-grade proteinuria. These findings suggest that anti-VEGF antibody use within 1 month may contribute to renal vulnerability during fruquintinib treatment. Mechanistically, renal toxicity associated with VEGF inhibition differs depending on the therapeutic target: ligand inhibition with bevacizumab is more commonly associated with thrombotic microangiopathy, whereas VEGFR tyrosine kinase inhibitors such as fruquintinib are associated with podocyte injury and glomerulopathies such as minimal change disease or focal segmental glomerulosclerosis. Therefore, prior exposure to bevacizumab may predispose patients to subsequent renal vulnerability, potentially contributing to the earlier onset and greater clinical impact of proteinuria observed in our cohort [19, 20]. Furthermore, emerging pharmacovigilance and molecular studies suggest that fruquintinib may induce proteinuria not only through VEGFR inhibition but also via off-target effects involving signaling pathways such as SRC and STAT3 [18]. These findings raise the possibility that fruquintinib-associated proteinuria may have drug-specific characteristics beyond class effects. Taken together, these findings suggest that proteinuria may have a substantial impact on treatment continuation in routine clinical practice and warrants careful monitoring and management.

Another clinically relevant finding of this study was that no clear difference in TTF was observed between the low- and high-RDI groups in the exploratory analysis. Reduced starting doses of fruquintinib have also been reported in other real-world cohorts, suggesting that upfront dose adjustment is a common strategy in routine clinical practice rather than an institution-specific approach [13–16]. However, previous real-world studies have generally described dose distributions without fully addressing how early toxicity-related dose modification and RDI may influence treatment continuation and outcomes. Population pharmacokinetic analyses have suggested that overall variability in fruquintinib exposure is relatively limited and that the effects of body weight and other covariates on exposure are generally less than 20%, which were not considered clinically meaningful [21, 22]. Notably, this degree of variability is close to the magnitude of a one-level dose reduction and may partly explain why appropriate dose modification did not appear to compromise treatment outcomes in our cohort. However, given the exploratory nature of the present analysis, these findings should not be interpreted as evidence that a reduced starting dose is equivalent to the standard starting dose. The clinical implications of reduced starting doses remain incompletely understood, particularly in frail patients who may be more likely to receive dose-reduced regimens in routine practice. In addition, whether a dose-escalation strategy starting from a lower dose could improve tolerability while maintaining efficacy has not been established for fruquintinib, although such an approach has been explored for other agents, such as regorafenib in the ReDOS study [4]. Future prospective studies should evaluate optimized starting-dose strategies using predefined eligibility criteria, baseline renal assessment, frailty or geriatric assessment, and protocol-specified dose-escalation or dose-modification rules. These findings suggest that AE-guided dose modification while continuing treatment may help to achieve a balance between treatment efficacy and tolerability in routine clinical practice.

From a clinical perspective, these findings suggest that early and careful monitoring of proteinuria is particularly important during the first cycle of fruquintinib treatment, especially in Japanese patients and in those with prior VEGF-targeted therapy exposure. Prompt recognition of proteinuria and timely dose modification may help maintain treatment feasibility while minimizing unnecessary treatment discontinuation in patients with mCRC.

This study has several limitations. First, this was a retrospective analysis conducted at only two institutions, and selection bias could not be excluded. In particular, the starting dose and dose modifications were determined at the discretion of the treating physician. Second, low-grade AEs may have been underreported because of the retrospective design. Third, OS data were still immature at the time of analysis, as the median follow-up period was relatively short, the upper limit of the 95% CI for median OS was NE, and only a limited number of patients remained at risk around the estimated median OS. Fourth, the RDI analysis included only patients who received fruquintinib for at least 2 months. This approach may have selected patients with relatively favorable early treatment courses and may limit the interpretation of comparisons between the low- and high-RDI groups.

## Conclusion

Fruquintinib demonstrated feasible effectiveness and manageable toxicity in Japanese patients with mCRC in routine clinical practice. Proteinuria was a key determinant of treatment feasibility and frequently led to early dose modification. Careful monitoring and early management of proteinuria may be important to optimize treatment continuation in clinical practice.

## Supplementary Information

Below is the link to the electronic supplementary material.

Supplementary Material 1

Supplementary Material 2

Supplementary Material 3

Supplementary Material 4

Supplementary Material 5

Supplementary Material 6

Supplementary Material 7

## Data Availability

The data presented in this study are available from the corresponding author upon reasonable request.
