# Supplementary material for: Proteinuria as the cause of early dose modification in Japanese patients with metastatic colorectal cancer treated with fruquintinib: a multicenter real-world study
Source: Int J Clin Oncol. 2026 Aug 27;31(11):2720–9. doi: 10.1007/s10147-026-03181-1 (PMC13710609; doi:10.1007/s10147-026-03181-1)
Supplement: Supplementary file 1 [file 10147_2026_3181_MOESM7_ESM.docx]

**Online Resource 1.** Dose modification patterns from treatment initiation to Day 29 and at treatment discontinuation.

The diagram illustrates changes in dosing from initiation to Day 29 (cycle 2) and to the last administered dose. The dose at Day 29 represents the dose at 29 days after initiation or the last administered dose in patients who discontinued treatment within 28 days.

**Online Resource 5.** Venn diagrams showing the overlap among proteinuria, hypertension, and hand-foot syndrome (HFS) for any-grade events and Grade ≥3 events.

**Online Resource 6.** Kaplan–Meier curves for (a) time to treatment failure (TTF) and (b) overall survival (OS) stratified by the worst grade of treatment-emergent proteinuria (≤ Grade 1 vs ≥ Grade 2). CI, confidence interval; HR, hazard ratio; OS, overall survival; TTF, time to treatment failure.

**Online Resource 7**. Kaplan–Meier curves for (a) time to treatment failure (TTF) and (b) overall survival (OS) stratified by the worst grade of hypertension (≤ Grade 1 vs ≥ Grade 2). CI, confidence interval; HR, hazard ratio; OS, overall survival; TTF, time to treatment failure.

**Online Resource 8.** Kaplan–Meier curves for (a) time to treatment failure (TTF) and (b) overall survival (OS) stratified by the worst grade of treatment-emergent hand-foot syndrome (≤ Grade 1 vs ≥ Grade 2). CI, confidence interval; HFS, hand-foot syndrome; HR, hazard ratio; OS, overall survival; TTF, time to treatment failure.
